# Maganin Gargajiya: Assessing the Benefits, Challenges, and Evidence of Traditional Medicine in Nigeria

**DOI:** 10.7759/cureus.71425

**Published:** 2024-10-14

**Authors:** Muhammed Raji Modibbo, Hadiza Ibrahim, Muzammil Y Sulaiman, Badir Zakir

**Affiliations:** 1 Department of Internal Medicine, Federal Medical Centre, Abuja, NGA; 2 Department of Internal Medicine, Zayed Military Hospital, Abu Dhabi, ARE; 3 Department of General Practice, Gwarinpa General Hospital, Abuja, NGA; 4 Department of General Practice, Childcare and Wellness Clinics, Abuja, NGA

**Keywords:** alternative remedies, complementary and alternative medicines, herb, nigeria, public health, traditional bone setting, traditional herbal medicine

## Abstract

In Nigeria, traditional medicine, commonly called "Maganin Gargajiya," holds a significant place in the healthcare system and is widely used due to its cultural relevance, accessibility, and perceived efficacy. This review seeks to evaluate whether traditional medicine is a net benefit or risk to public health. It will explore the pros, such as the care provided by traditional healers, and the cons, including delays in seeking conventional treatment and the potential health risks associated with unregulated herbal remedies. This article synthesizes existing studies on traditional medicine in Nigeria, evaluating both the benefits and risks associated with its use, based on secondary data analysis. The review aims to provide a balanced perspective on the role of traditional medicine in Nigeria, examining whether it should be integrated into, regulated alongside or divorced completely from modern healthcare to improve overall patient outcomes and safety.

## Introduction and background

Traditional medicine, colloquially known as "Maganin Gargajiya" in many parts of Nigeria [1,2], is a term used for any form of healthcare delivery outside the confines of a hospital. The WHO defines traditional medicine as "the sum total of the knowledge, skill, and practices based on the theories, beliefs, and experiences indigenous to different cultures, whether explicable or not, used in the maintenance of health as well as in the prevention, diagnosis, improvement or treatment of physical and mental illness" [3]. Complementary and alternative medicines are terms that encompass health practices that are not integrated into the mainstream healthcare system of a country and are often used interchangeably with traditional medicine [3]. Traditional medicine includes the use of herbs and plant-based preparations, known as herbal medicines, as well as practical procedures like traditional bone setting [3,4]. Historically, traditional healing has been an integral part of Nigeria's health system, predating the colonial era [5]. Historical shifts, such as colonial influence and the introduction of Western medicine, have significantly shaped perceptions of traditional medicine in Nigeria, leading to a complex relationship where it is often seen as a primary healthcare source in rural regions and a complementary option in urban settings [5]. Over time, the practice began to decline due to government restrictions and the rise of Western medicine in the country. It has recently experienced a resurgence in popularity as traditional healers offer a holistic blend of physical, mental, and spiritual care [5]. Most Nigerians rely on some form of traditional medicine [6]. Current estimates suggest that over 80% of the general Nigerian population uses some form of traditional medicine [6]. The factors contributing to its widespread use are its easy accessibility, ready availability, affordability, convenience, and the public perception of its safety and efficacy [6,7]. Traditional healers are also highly trusted in rural communities [7].

Given its widespread use, two key questions arise: Are traditional healers and traditional medicine beneficial or harmful? Should the government clamp down on these practices or incorporate them into the Nigerian healthcare system? On the one hand, traditional healers in Nigeria sometimes play a crucial role in managing minor health conditions, offering care for some ailments, and referring patients to modern health facilities as needed [7]. Their unique approach and the immense trust people place in them highlight their significant role in healthcare. So, there is considerable potential for integrating traditional and contemporary practices to enhance patient care and outcomes [7]. On the other hand, while traditional healers can offer certain benefits, they pose significant health risks. Evidence has shown that many herbal drugs contain high levels of dangerous metals [8]. Additionally, individuals who use alternative medicine often neglect prescribed medications [8] and often frequently delay seeking appropriate expert medical attention [9,10].

With that in mind, this paper aims to answer the above questions. By examining the literature, we will present the case for the prosecution and the defense of "Maganin Gargajiya" in the Nigerian context. This paper will analyze the benefits, risks, and barriers preventing the wider implementation of traditional medicine in contemporary healthcare practices in Nigeria.

## Review

Harms of traditional medicine

**Figure 1:**
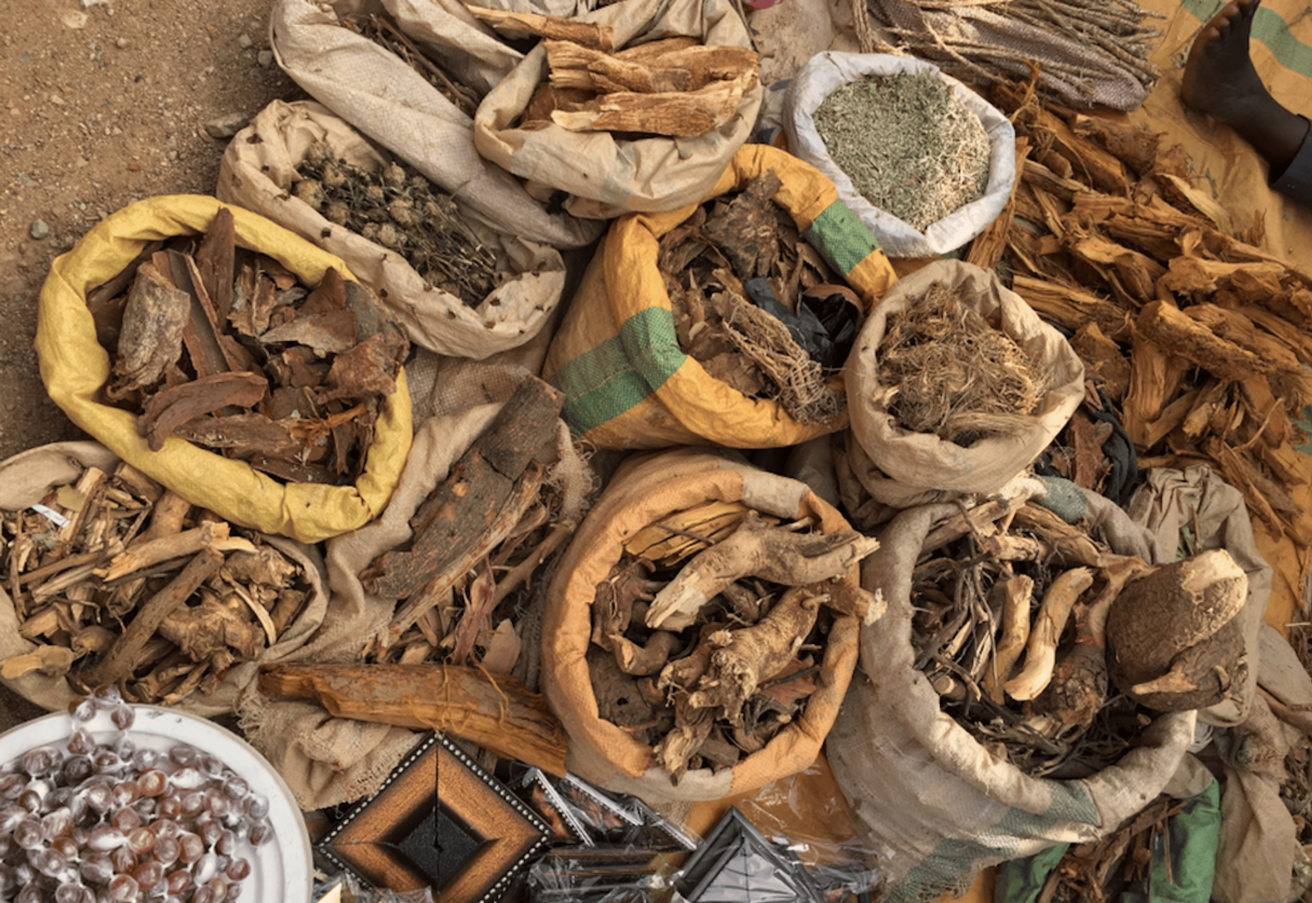
A view of Hausa traditional medicines Image credits: Image ©2021 Musa Vacho77, licensed under Creative Commons Attribution-Share Alike 4.0 International License [15]

Research into herbal medicine in Nigeria raises significant safety concerns, including bodily harm, potential contamination, harmful drug interactions, and adverse health effects. Ensuring the safety of these traditional remedies and practices is crucial for maintaining societal health. One investigation found that most herbal medicine samples contained elevated levels of heavy metals, including cadmium, lead, and mercury, all recognized as toxic and potentially harmful [8]. Another study corroborates this finding, which noted that contamination with heavy metals in herbal medicines is often due to soil pollution where the plants are cultivated [11]. Additionally, research has shown that microbial contamination levels in these herbs exceed the acceptable limits set by the World Health Organization [8]. A separate analysis of 210 herbal medicine samples from Nigeria identified various aflatoxins at different concentrations, with an average concentration of 18.6% [12]. This highlights a concerning level of aflatoxin contamination in herbal remedies, given that aflatoxins are known carcinogens [13]. Ezuruike and Prieto demonstrated that certain plants (Figure 1) used as herbal drugs can cause organ toxicity, particularly affecting the kidneys and liver. Their research also revealed that several plants affect P-glycoprotein and/or cytochrome P450 enzymes, which are essential for drug metabolism. Furthermore, many other plants were found to alter glutathione levels, potentially influencing the pharmacokinetics of concurrently administered prescription medications [14].

**Figure 2:**
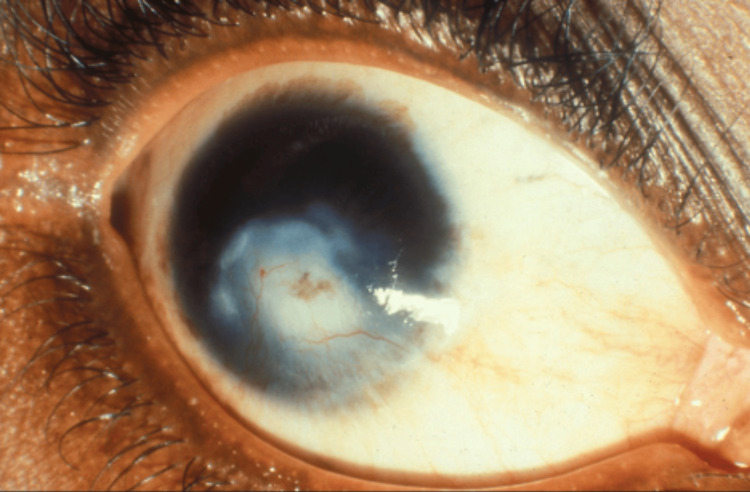
Corneal scarring in a young woman due to traditional medicine used when she was a child Image credits: Image ©2013 Hans Limburg, licensed under Creative Commons Attribution-NonCommercial 2.0 International License [18]

A cross-sectional study involving 794 participants in northern Nigeria found that 64% used traditional herbs as aphrodisiacs. Regarding safety, 20.7% of participants reported experiencing side effects, and 3.9% required hospitalization due to severe side effects of these herbs [16]. In the context of managing wounds, local herbs have been linked to abnormal wound healing, exacerbating skin damage and increasing the risk of keloid formation [17]. This issue was highlighted in a case report of a 52-year-old immunocompetent man with herpes zoster ophthalmicus published in the Nigerian Journal of Clinical Practice. Traditional topical remedies have also been reported to cause corneal scarring (Figure 2), ocular damage, and even potential blindness in Nigeria [9].

In addition to their toxic effects, traditional practices can cause harm in other ways, as illustrated by a case series of patients who suffered severe burns due to these practices. These cases underscore how certain traditional methods, prevalent in the local environment, can lead to significant injury and morbidity [19]. Traditional bonesetting is an ancient practice involving manually manipulating joints and bones to treat musculoskeletal injuries [20]. Traditional bonesetters (TBS) have always been a significant part of Nigerian healthcare, with most fracture patients consulting them before seeking hospital care [21]. However, TBS practices often lead to serious orthopedic complications, ranging from minor issues like limb length discrepancies to severe conditions such as limb gangrene, blisters, pressure sores, Volkmann's ischemia, and crush syndrome [21-24].

Besides safety concerns, alternative medicine also faces issues related to unethical practices among many traditional healers [25]. Some practitioners engage in unprofessional and unacceptable behaviors, neglecting core healthcare principles. These violations include exploiting vulnerable patients, disregarding their well-being, and prioritizing profit over proper care [25]. Such unethical conduct not only jeopardizes individual health but also undermines the integrity of traditional medicine.

There are also questions about the efficacy of alternative medicine as practiced in Nigeria. A study conducted at the University of Nigeria Teaching Hospital, Enugu, found that most patients using traditional medicine did not experience any benefits [26]. Many reported adverse effects, including disappointment and health complications [26].

Potential benefits of traditional medicine

**Figure 3:**
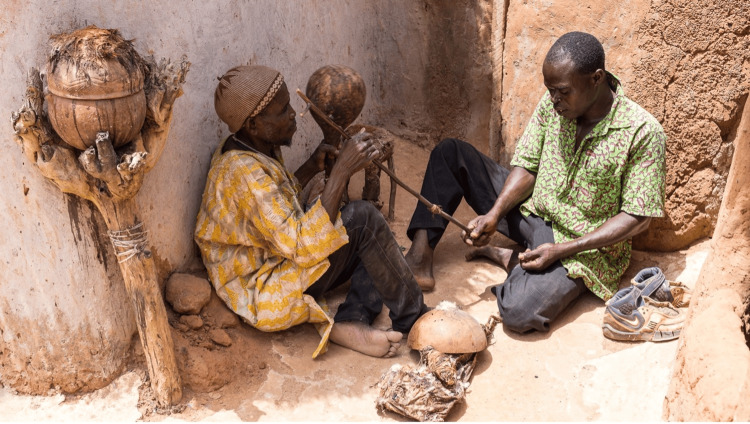
Consultation between a traditional healer and a patient Image credits: Image ©2016 William Haun, licensed under Creative Commons Attribution-NonCommercial 4.0 International License [27]

A large study conducted at Noma Children’s Hospital in Sokoto evaluated the role of traditional healers in managing patients with noma (cancrum oris) [7]. The study found that some traditional healers could recognize the early stages of noma. Given that these healers are often the first point of contact for most of the rural population (Figure 3), their ability to detect and refer cases could be life-saving [7]. Based on these findings, the researchers believe that with additional training, traditional healers could significantly contribute to the early recognition and prompt referral of patients requiring urgent medical attention [7].

In addition, research has demonstrated that several herbal medicines possess antimicrobial properties. Plants such as moringa (*Moringa oleifera*), neem (*Azadirachta indica*), and guava (*Psidium guajava*) have shown efficacy in inhibiting microbial growth [28]. These findings highlight the potential of these medicinal plants in treating various health conditions, supported by studies validating their effectiveness [28]. Furthermore, some herbal drugs have proven effective for malaria treatment, with many identified plants demonstrating antimalarial activity [29]. While some African medicinal plants have shown promising antitrypanosomal effects, further empirical evidence is needed to fully assess their potential [30]. In addition to their general antimicrobial properties and potential to treat systemic conditions, herbal remedies have also shown specific benefits in dermatological applications. Research conducted at the Dermatology Clinic, Lagos State University Teaching Hospital, suggests that herbal remedies have been beneficial for certain skin conditions, as evidenced by their effective application in treating various issues, including eczema, seborrheic dermatitis, impetigo, tinea capitis, scabies, and other conditions [31].

Barriers to integration

Despite the promising evidence of the efficacy of herbal medicine, the practice still faces major challenges related to documentation and clinical validation [28]. The lack of standardized documentation and comprehensive data hinders any attempts at widespread adoption of herbal treatments in clinical practice [28]. Therefore, while herbal medicine has potential benefits and immense historical significance, there is currently insufficient clinical evidence and modernization to support its broader use in mainstream healthcare [28,32]. This gap in evidence and standardization poses a serious obstacle to integrating herbal medicine into conventional healthcare systems.

## Conclusions

In conclusion, while traditional medicine plays a significant role in cultural practices and offers a range of potential benefits, its integration into contemporary health systems must be approached with caution and regulation. The prevalence of toxic traditional medicines highlights the urgent need for government intervention to address harmful substances and protect public safety. It is also vital to address contamination of traditional herbs and unethical practices among traditional healers to enhance the safety of traditional medicine in Nigeria. In addition, investment in researching medicinal herbs is crucial to uncovering their therapeutic potential and providing solid clinical evidence supporting their use.

Furthermore, regulating traditional practices should be a priority to minimize risks and uphold safety standards. Complementing these regulatory efforts with targeted awareness campaigns will help educate the public about the dangers of harmful traditional practices. Additionally, investing in the training and education of traditional healers, to integrate them into the primary health system, could foster a more comprehensive and safer approach to healthcare. Implementing these measures can bridge the gap between traditional and modern medicines, creating a healthcare environment that honors cultural practices while prioritizing patient safety and efficacy.
